# Essential elements of and challenges to rapid ART implementation: a qualitative study of three programs in the United States

**DOI:** 10.1186/s12879-022-07297-3

**Published:** 2022-03-31

**Authors:** Kimberly A. Koester, Lissa Moran, Noelle LeTourneau, Lyndon VanderZanden, Susa Coffey, Pierre-Cedric Crouch, Janessa Broussard, John Schneider, Katerina A. Christopoulos

**Affiliations:** 1 Department of Medicine, University of California, San Francisco, 550 16th Street, San Francisco, CA USA; 2 Department of Medicine, Zuckerberg San Francisco General Hospital, University of California, San Francisco, San Francisco, CA USA; 3 Howard Brown Health Center, 4025 N. Sheridan Rd, Chicago, IL USA; 4 San Francisco AIDS Foundation, 470 Castro Street, San Francisco, CA USA

**Keywords:** HIV treatment, Rapid ART, Immediate ART qualitative methods

## Abstract

**Background:**

Antiretroviral therapy (ART) initiation on the day of an HIV diagnosis or as soon as possible after diagnosis, known as rapid ART (henceforth “RAPID”), is considered to be a safe and effective intervention to quickly reduce viral load and potentially improve engagement in care over time. However, implementation of RAPID programming is not yet widespread. To facilitate broader dissemination of RAPID, we sought to understand health care worker experiences with RAPID implementation and to identify essential programmatic elements.

**Methods:**

We conducted 27 key informant interviews with medical providers and staff involved in RAPID service delivery in three distinct clinical settings: an HIV clinic, a Federally Qualified Health Center and a sexual health and wellness clinic. Interviews were structured around domains associated with the Consolidated Framework for Implementation Research and were audio-recorded, transcribed, and thematically analyzed.

**Findings:**

We identified seven (7) essential elements across settings associated with successful RAPID program implementation. These high-impact elements represent essential components without which a RAPID program could not function. There was no one requisite formation. Instead, we observed a constellation of essential elements that could be operationalized in various formations and by various people in various roles. The essential elements included: (1) presence of an implementation champion; (2) comfort and competence prescribing RAPID ART; (3) expedited access to ART medications; (4) expertise in benefits, linkage, and care navigation; (5) RAPID team member flexibility and organizations’ adaptive capacity; (6) patient-centered approach; and (7) strong communication methods and culture.

**Conclusions:**

The RAPID model can be applied to a diverse range of clinical contexts. The operational structure of RAPID programs is shaped by the clinical setting in which they function, and therefore the essential elements identified may not apply equally to all programs. Based on the seven essential elements described above we recommend future implementers identify where these elements currently exist within a practice; leverage them when possible; strengthen them when necessary or develop them if they do not yet exist; and look to these elements when challenges arise for potential solutions.

**Supplementary Information:**

The online version contains supplementary material available at 10.1186/s12879-022-07297-3.

## Background

Advances in the treatment of HIV have evolved substantially over the last three decades. Consistent use of antiretroviral therapy (ART) allows individuals to achieve and maintain optimal health, while rates of onward HIV transmission are virtually eliminated when viral load is durably suppressed [1–3]. Healthcare delivery systems must similarly evolve to fully realize the benefits of HIV treatment advances. This includes identifying effective strategies to meaningfully link and retain individuals in care in the immediate and longer term.

Early initiation of ART confers clinical and public health benefits [4–9]. ART initiation on the day of diagnosis or as soon as possible after diagnosis, known as rapid or immediate ART (henceforth “RAPID”), is a safe and effective strategy to quickly reduce viral load and potentially improve engagement in care over time [9–11]. RAPID has been endorsed by each of the international and national health organizations charged with developing guidelines on HIV management [13–16]. Implementation of RAPID programming, while growing, is not yet widespread. While RAPID programming can be resource-intensive, studies show that it is effective in achieving rapid initiation of ART and reduced time to virologic suppression [12, 17–19]. Just how resource-intensive is an open question, and key questions about optimal implementation of RAPID remain.

Researchers have called for studies assessing how RAPID (as a collection of immediate linkage and medication initiation services) is operationalized in different U.S. healthcare settings [20, 21]. Understanding how distinct clinical settings operationalize the component parts of RAPID—i.e., immediate access to medical and psychosocial assessment, assistance with establishing insurance benefits, intensive HIV counseling and education, ordering of baseline laboratory tests, provision of ART medications and linkage to HIV primary care services—could be critical to the widespread scale-up of programs [18, 19, 22]. Providing an assessment of the types of implementation strategies that accelerate access to ART and retention in care will also move the field forward.

To that end, we conducted multi-site qualitative research to assess RAPID program implementation efforts from the perspective of providers and staff. Our work provides evidence on the necessary resources and systems-level transformations needed to adopt the RAPID model in a variety of clinical settings. The purpose of this article is to outline the essential elements and challenges of RAPID implementation across three distinct settings.

## Methods

### Study design and settings

This paper reports on a series of studies unified by a focus on assessing RAPID implementation processes. Initially, we designed a qualitative study to understand provider/staff perspectives on RAPID implementation in the San Francisco-based HIV clinic that first pioneered RAPID in the United States [20]. We subsequently added two sites in order to study adaptations to the original model. Table 1 depicts the organizational characteristics of each setting. Briefly, the HIV clinic (henceforth Innovator) began implementing RAPID in 2013. Inspired by the success of the original RAPID model, a federally qualified health center (henceforth FQHC) in Chicago launched their own program based on the San Francisco model in 2018. Also in 2018, a robust HIV testing program situated within a sexual health and wellness clinic (henceforth Testing Site) in San Francisco launched its own RAPID program. As a setting that did not provide primary care, this particular RAPID model was distinct in that they started clients on ART and then linked them to an HIV care provider for ongoing care.

**Table 1:** Study site characteristics

Site characteristics	Innovator site	FQHC	Testing site
Clinic type	Academic HIV clinic in safety-net hospital system	Federally Qualified Health Center	Sexual health clinic and testing site
RAPID patient source	Combination of patients diagnosed w/in hospital system & off site	Combination of patients diagnosed on and off site	Majority of patients diagnosed on site
RAPID visit structure	By appointment & drop-in	By appointment & drop-in	By appointment & drop-in
RAPID Team	Prescriber (MD, NP) + RN + MSW	Health Educator (HE), RN or MA + Prescriber (MD, DO, NP, PA, APN) + Linkage to Care Coordinator (LTC) + Pharmacist	Prescriber (NP) + Health Navigator
Composition of RAPID Visit	Typically, patient seen by whole RAPID team at once	Typically, patient seen by RN, HE, or MA, then LTC, then Prescriber	Typically, patient seen by Health Navigator and NP only
Linkage to HIV Primary Care	HIV primary care on-site (no external linkage)	Within FQHC system (linkage to internal primary care at one of 10 clinics)	Off-site (linkage to external primary care)
# of RAPID 2019 Encounters	41	198	70
# of Patients w/HIV	2800	4942	n/a
# of HIV Providers	38	54	7
# of Patients/clients	n/a	30,013	9600
# of Providers	n/a	63	7

### Sampling, recruitment and data collection procedures

At each site, providers and staff who ran or oversaw the program facilitated identification of prospective key informants. To be eligible for the study, providers and staff had to be involved in RAPID service delivery. Authors KK or LM reached out to potential key informants via email to invite them to join the study, using a standard script and offering to schedule an interview at a mutually convenient time. Depending on logistics, such as the availability of a private space to conduct an interview and the location of the key informant, interviews at the sites in San Francisco were either conducted over the phone (2) or in-person (19). All interviews conducted with FQHC key informants were conducted over the phone due to the geographic distance between the San Francisco-based research team and the FQHC site. Both KK and LM are qualitative researchers with extensive experience building rapport and conducting in-depth interviews with HIV providers/staff. KK and KAC developed the interview guide with domains associated with the Consolidated Framework for Implementation Research (CFIR) in mind: intervention characteristics, inner and outer settings, individual characteristics, and implementation process [23]. The interview guide is available as Additional file 1. The CFIR’s multi-level and -dimensional implementation considerations defined the scope of the interview guide and thus the universe of the data we collected. However, our findings were ultimately scattered across multiple domains and constructs, and thus the CFIR was not a useful framework for presenting our analysis. (See Additional file 2 for an illustration of interview questions and findings mapped onto the CFIR for reference.) Interview domains included pre-implementation and implementation experiences, attitudes about RAPID, and patient experiences. Interviews lasted up to 60 min, were audio-recorded, and transcribed verbatim. Each key informant was offered a $20 incentive as a token of appreciation.

### Data analysis

After each interview, KK or LM drafted a fieldnote to capture a synopsis of the interview content, changes to the interview guide, emerging themes, and any additional information related to the interview that would not be captured in the transcript. These fieldnotes were distributed to the broader team and discussed during meetings. Initially, our descriptive analysis consisted of a deductive examination of the implementation experiences at the Innovator Site. Authors KK and LM reviewed the fieldnotes and transcripts associated with all the interviews from that site and drafted analytic memos based on each interview. These memos were compared across informants and an overall memo depicting key themes related to the implementation process, essential elements, and challenges was developed for the Innovator Site. This summary informed modifications to the guide used during interviews with key informants in the Testing and FQHC sites. It was eventually used as a foundation to compare and contrast interview data from each of the other RAPID programs.

As we accumulated more data, we modified our analytic strategy to include coding. We also integrated an additional person to our analytic team (NLT). NLT was the data manager for the study and had prior experience with qualitative research; her familiarity with the quantitative data being collected for the study offered a unique perspective to the analytic team. The three analysts collectively developed a codebook following a process authors KK and LM have successfully used in the past. This process included reading a transcript selected for its data richness aloud to discuss, interpret and collectively generate a list of preliminary codes [24]. Once a preliminary list of codes was drafted, we independently coded 2 additional interviews and met to cross-check our coding choices. We further refined the codebook by elaborating on meanings and dropping or adding codes. The remaining interviews were divided across the team, with each transcript assigned to a primary coder and a secondary reviewer. Once the data were coded, verified and entered into Dedoose [25], the team developed a data reduction plan. Our chief goal during the analysis process was to compare and contrast across cases (interviewees) within a site and then to expand comparison across sites. This allowed us to identify similarities, differences and deviant or negative cases. We generated code reports within Dedoose for 13 codes (codebook available upon request) to read and summarize. During bi-weekly data reduction sessions, we documented our collective interpretations and analytic insights. These data summaries were the basis for our identification of essential elements and implementation challenges presented below. We held a study report back session (member check) to share our preliminary findings with a subset of our key informants [26]. That session included providers and staff from the Innovator site and sensitized our team to the findings that were thought to be maximally useful to the field, such as emphasizing the importance of starter packs. This process also led to some minor but important adjustments in the representation of some aspects of the programs, such as discouraging our colloquial use of the term “triage” in the context of RAPID encounters.

### Findings

We conducted 27 interviews from March 2018 to February 2020 (see Table 2 for participant demographics). We identified seven (7) essential elements across settings associated with successful RAPID program implementation. We provide examples of the application of these essential elements and indicate if and how they functioned differently in each setting (see Table 3). We also highlight ongoing operational challenges to inform future implementation efforts elsewhere.

**Table 2:** Participant demographics

N = 27	N (%)
Gender	
Male	10 (37%)
Female	15 (56%)
Trans/non-binary	2 (7%)
Race/ethnicity	
White	19 (70%)
Latinx	6 (22%)
Asian	1 (4%)
Black	1 (4%)
Years in HIV	
< 5	9 (33%)
5 to 15	10 (37%)
> 15	8 (30%)
Role within RAPID program	
Prescribing provider (MD, NP, PharmD)	8 (29%)
RN	4 (15%)
Social work/navigator/linkage specialist	11 (41%)
Leadership + prescribing provider	4 (15%)

**Table 3:** Essential elements of RAPID, operationalized by site

	Innovator site	Testing site	FQHC
Comfort and competence prescribing ART	MDs/NPs prescribing HIV primary care team w expertise in HIV care Select cadre of RAPID providers from broader pool of primary care providers	NPs prescribing (recent addition for other prescribing services at site) All providers available to participate in RAPID as any other sexual health service	MDs/NPs/DOs/PAs/APNs prescribing HIV primary care team w expertise in HIV care All providers available to participate in RAPID as any other sexual health service
Expedited access to ART	Starter packs + Rx for ongoing ART to be filled by patient	Starter packs + Rx Proximity to pharmacy for immediate prescription fulfillment (same-day Rx fulfillment so successful, starter packs rendered unnecessary)	Prescription that could be filled immediately or next-day at the on-site pharmacy Proximity to pharmacy for immediate prescription fulfillment Medication sample packs
Benefits, linkage, and care navigation	Access to patient drug assistance programs (same day ADAP) Clinic receives warm hand-offs from referrals; on-site HIV primary care, occasional external linkage necessary Social worker as part of RAPID team to assess and connect pt to wraparound services Navigation of pt through RAPID process (“red carpet” treatment)	Access to patient drug assistance programs (ADAP) Ability to assess eligibility and enroll patients in benefits programs same-day Assess for additional needs (housing, mental health, etc.), psychosocial support on site Off-site linkage to external HIV PC provider (includes assessments of benefits and needs to find appropriate match, coordinating transition)	Access to patient drug assistance programs (ADAP) Insurance and patient assistance program knowledge for patients to access free meds Clinic receives referrals as warm hand-offs and self-referrals; on-site HIV primary care, no external linkage necessary Assess for additional needs (housing, mental health, etc.), psychosocial support on site Navigation of pt through RAPID process, ensuring warm hand-off to internal HIV PC provider
Flexibility and adaptive capacity	Appointment & Drop-in Interdisciplinary Team Prescribing clinicians flexible in accommodating RAPIDs in the schedule Iterative program development and improvement	Appointment & Drop-in Interdisciplinary Team Iterative program development and improvement (i.e., cross-training benefits navigators and health navigators; expanding physical space for RAPID; opening access to mental health services at RAPID visit when needed)	Appointment & Drop-in Interdisciplinary Team Iterative program development and improvement (i.e., adding Uber account, adding on-site linkage coordinator to clinic)
Patient-centered approach	Services for patients experiencing homelessness, substance use RAPID as a red-carpet event to provide patient support Warm hand offs between RAPID team members	LGBTQ + community focus, including transgender and gender nonbinary services Community outreach and site-specific access (for patients experiencing homelessness), mobile testing units Meet with as few people as possible during encounter Attitude of doing whatever it takes to meet the patient’s need	LGBTQ + community focus, including transgender and gender nonbinary services Services for patients experiencing homelessness, mental health needs, substance use Community outreach and testing Meet with as few people as possible during encounter, stays in same exam room throughout process, (when possible/desirable) has prescription brought to them Attitude of doing whatever it takes to meet the patient’s need Accessing all needs for patients (i.e., clothing, food, transportation vouchers) In-house linkage to minimize time patient spends alone
Communication methods and culture	Interdisciplinary team, team members present and together for some parts of the RAPID encounter Central pager system	Small team communicates informally throughout daily activities	Centralized “linkage to care phone” Repeated check-ins among RAPID team throughout RAPID process, and continued in-person and EMR-based communication throughout full linkage to care process (first 6–12 months of patient’s care) RAPID team members use EMR to read patient notes during RAPID encounter

### Essential elements for RAPID program implementation

Key informants from each site detailed barriers and facilitators to successful implementation of RAPID. From these, seven high-impact elements emerged that represent essential components without which a RAPID program could not function. There was no one requisite formation; instead, we observed a constellation of essential elements that could be operationalized in various formations and by various people in various roles. The essential elements included: (1) presence of an implementation champion; (2) comfort and competence prescribing RAPID ART; (3) expedited access to ART medications; (4) expertise in benefits, linkage, and care navigation; (5) RAPID team member flexibility and organizations’ adaptive capacity; (6) patient-centered approach; and (7) strong communication methods and culture.

1. *Implementation champion*

The health services implementation science literature supports the importance of a champion in quality improvement and implementation efforts [27–29]. Among the sites in this study, each had one or more individuals who served as a champion invested in the success of the RAPID intervention. In the case of the Innovator Site, the champions were in positions of leadership and instructed key staff to offer patients ART on the day of diagnosis. One interviewee recollected thinking that prior to embarking upon RAPID program implementation, it would be “impossible” to have a patient start on medications on the day of disclosure. She then explained how quickly that “impossible” mindset changed:

“[I]t wasn’t a proposal. It was an order. … When [Champion’s name] came and said, ‘We're going to launch a new initiative and we're actually going to try to start people on the day of diagnosis,’ we actually thought she was crazy. …. We thought it was just impossible that the barriers - the ability to get a patient to start medication could not be merged in the disclosure of a new diagnosis. … [But the were] the boss … and so we said, ‘Okay. We'll try it,’ but we weren't very confident that it could work. Within the third day or fourth day of the launch of RAPID, we had done, probably maybe three or four RAPID cases. Our team just - at the end of the day, informally, all found ourselves in the same room and was sharing the experiences that we'd had with these new diagnoses and were all completely blown away.” (Innov KI01)

The champions in the Testing and FQHC sites were motivated by the success of the Innovator Site and were eager to have patients realize the benefits of RAPID ART. Collectively, RAPID champions drove the implementation process forward and were instrumental in overcoming challenges that might have otherwise derailed successful implementation.

“[Champion’s name] is the biggest visionary in this field, and it's simply down to him. He was the one who initiated this, and that's the end of it... He was so motivated to do this. … And initially, obviously, it was a little confusing as to what bloods to order, what are the different scenario, et cetera, but now everything is built into the template. … [Champion] is the biggest force behind this, to me.” (Test KI06)

(2) Prescribing: at least one individual on the team possesses comfort and competence in prescribing same-day ART

As a medical intervention*,* a RAPID program requires a provider with prescribing privileges to initiate immediate ART for newly diagnosed patients on an as-needed basis, and a willingness to do so. Key informants from the sites that provide primary care (Innovator and FQHC) indicated that some prescribers were well-suited to conducting a RAPID visit because they felt comfortable prescribing ART in the absence of initial laboratory results and because they were amenable to the disruption of drop-in visits. In contrast, discomfort with the disruptive nature of RAPID prevented some providers from being involved in RAPID encounters as explained below.

“I knew some providers would say no to a RAPID appointment because we can't ever plan for them…. We have to sort of drop everything we're doing … and we knew specific providers that were open and willing and excited to do that. ..we would just have a mental list of people who were willing and open to doing those appointments. So, I think that it as a practice and as a protocol was accepted, but not every practitioner felt comfortable doing them.” (Innov KI05)

Within the Testing Site, implementing RAPID was made possible because they had recently expanded their workforce, adding nurse practitioners (NPs) to provide hepatitis C treatment and HIV pre-exposure prophylaxis (PrEP) as new services. Prior to that, they did not have staff with prescribing privileges in their nurse-led clinic. Notably, services were offered in response to a perceived gap in services, thus interviewees affiliated with the Testing Site expressed excitement about and comfort with offering RAPID ART: “I don’t think there’s a single one of us that I know of that is not behind this.” (Test KI01).

Click or tap here to enter text. In addition to basic competencies in HIV care, RAPID required provider acceptability—belief that the model was beneficial for patients, that it would not do patients harm, and that the value of immediate ART outweighed the risks of starting ART before initial laboratory results (i.e., genotype, HIV RNA, CD4 and renal function) were available.

“I’d rather err on the side - ‘cause worst case scenario, you get them the pills, they take one, and then you never see them again—they don’t take anymore. And like, they’re not going to cause themselves that much harm from it. I would be horrified, horrified if I did not give a treatment to somebody who would’ve successfully done it. I’d rather … err, whatever else, giv[e] someone the opportunity who maybe wasn’t ready than to remotely risk someone not getting care.” (Test KI04)

The quote above illustrates this prescriber’s risk–benefit calculation. Notably they expressed confidence in RAPID as an intervention to facilitate retention in care. Key to the success of RAPID programming was identifying at least one provider with the ability to prescribe medication, who had the comfort and confidence in the RAPID model and in their own knowledge of HIV care and treatment to prescribe ART at the time of diagnosis.

(3) Expedited access to ART medications.

Accessing ART within an expedited timeframe was identified as a discrete competency, one that involved expertise in insurance eligibility assessment, payer systems, and funding mechanisms, such as same-day ADAP enrollment or pharmaceutical programs that offer copay cards. Our Innovator Site, for example, relied on “starter packs” of ART (purchased by the clinic) in addition to a prescription, so all patients could start ART while ADAP and Medicaid eligibility was established. The FQHC site took a similar approach, but utilized pharmacy access programs and pharmaceutical samples, rather than starter packs. RAPID patients at the FQHC site were provided a prescription for immediate or next-day fill at an on-site or local pharmacy, and if necessary, were enrolled in a pharmacy access program or given pharmaceutical samples if they would have otherwise had to delay initiation until ADAP and Medicaid enrollment was completed. The Testing Site had multiple access points to expedited access to ART medications, such as starter packs and same-day pick-up at nearby pharmacies, which could be tailored to a patient’s need and around a variety of logistical or administrative constraints.

“They leave with five days of meds. So, it's not just like, "Okay, here's this prescription," because there's still 1,000 things that can go wrong between, like, handing them a prescription and picking up that prescription from your, you know, local, unfriendly Walgreens, and so …Yeah, starter packs are amazing.” (Innov KI04)

As a high-volume PrEP provider, the Testing Site had a lot of experience with and clear processes in place to facilitate efficient access to PrEP medication. Staff transferred these skills over to facilitate expedited access to ART.

“We [had] been doing PrEP. We got really, really good at being able to get any med for anyone, like super easy. … We were already doing that, so we already had the access to getting meds part down pat.” (Test KI04)

(4) Benefits, linkage, and care navigation.

Benefits, linkage, and care navigation represent a suite of essential skill areas for RAPID operation. Benefits navigation encompassed all activities associated with eligibility assessments and enrollments into benefits, including health insurance, drug assistance programs like ADAP, and access to support programs. While expedited access to ART medications (finding #3) pertains to the immediacy of medication access on or near the day of diagnosis, benefits, linkage, and care navigation pertain to setting a client up with medications, primary care, and wraparound services going forward. RAPID support staff who could quickly and competently navigate insurance and funding mechanisms provided critical support to patients. A Testing Site informant explained the crucial role played by a “dedicated benefits team”:

“Having the nurses, well trained in sexual health and HIV treatment is definitely one of the most [important] things that made this program successful, and also having a dedicated benefits team. Without that you can have the best nurse that's well versed in all aspects of HIV treatment, but if they're not well versed in how to get the patient the medications…. It's just gonna end right there. Yes, you have great care, but can you afford your medication when you leave the clinic? No.” (Test KI09)

Same-day access to insurance and benefits programs provided the material benefits of an ART prescription and on-going HIV care services for low- or no-cost to patients, as well as psychosocial support to patients for whom concerns about paying for care and treatment were a barrier. An FQHC interviewee spoke about the importance of communicating about insurance options to the patient early on to reduce anxiety and/or misperceptions related to the cost of HIV care and treatment.

“… the number one question I usually get from people when I talk to them, is how much is this all going to cost me? So being able to say confidently that we have the resources to get you all of this for free, I think is a big relief for patients, and a big relief in why they're able to initiate that.” (FQHC KI05)

Care navigation included identifying and striving to meet a patient’s emotional and material needs during a RAPID visit. Across the sites, care navigation was performed by multiple service providers with different titles, among them patient or health navigator, social worker, nurse, linkage coordinator, and case manager. The person or persons in the care navigation role typically oversaw and facilitated a patient’s movement through the RAPID process, assessing additional needs (housing, mental health care, etc.), providing psychosocial support, and either physically escorting the patient from one provider station to another (exam room, benefits counselling, lab, pharmacy) or serving as the patient’s single point of contact while other services were completed or procured behind the scenes.

Prior to initiating RAPID programs, these clinics emphasized linking a newly diagnosed individual to care where treatment would presumably be initiated. The current emphasis allows newly diagnosed individuals to initiate ART first and then be linked to ongoing HIV primary care either within the same health center or to an external HIV primary care. Implementing RAPID allowed staff in these sites to slow down the linkage process, thus creating the opportunity to get to know a patient and make a more intentional linkage to care connection. One key informant from the Testing Site talked about how removing the urgency to get an individual quickly linked to a primary HIV care site allowed the health navigator to re-direct their attention to, among other things, tending to the emotional state of the client. Test_KI03 explained:

“Sitting with the client to me is one of the most important things, and establishing some rapport so that they can become increasingly comfortable to work with me and share some information that helps me figure out what's an appropriate medical home for them. … So having someone start on RAPID is invaluable because it gives us that breathing room. We don't have to, like, oh, my God, oh, my God, oh, my God, they have to start on medication, they have to, you know, get into care yesterday. But they're still sitting there shaking. We haven't even started the intake process, let alone, you know they don't have medical insurance and, you know, just all these variables. So it's just invaluable.” (Test KI03)

- (5) RAPID team member flexibility and organizations’ adaptive capacity.

Providing RAPID services was, to varying degrees, disruptive in each setting. Success in these clinics was due in large part to each site having sufficient adaptive capacity or the ability to adapt to changes in the organization’s environment. While there was no way to predict when an individual would receive a positive HIV test result, all sites planned for RAPID service-related disruptions. Each site demonstrated flexibility and adaptive capacity differently; however, they shared two key components: each included capacity for drop-in visits; and each site engaged in an iterative process of feedback and correction to modify and refine their programs.

The drop-in element, and a clinic process built to absorb a certain amount of unpredictability, were crucial. The clinic scheduling system did not need to be primarily drop-in, but having a drop-in component at minimum—i.e., drop-in hours or appointment slots reserved for drop-ins—was essential for the flexibility to take on unscheduled appointments.

“I’d say more than half of our appointments are drop-in. It's like an open-access model, and that’s where you get to squeeze in, you know, PrEPs, the PEPs [Post-exposure prophylaxis], the iARTs [ART re-engagement], the RAPIDs.” (Test KI08)

Many informants described a similarly flexible and adaptive approach to RAPID implementation and quality improvement. Two sites implemented RAPID without engaging in formal pre-implementation activities such as engaging stakeholders and identifying barriers. The remaining site spent considerable time planning for and identifying potential barriers. They predicted that more linkage staff might be needed, however, confirmation of this prediction would need to be made after the program was initiated. Furthermore, no informants described personal involvement in a formal RAPID-related quality improvement protocol once implementation began. RAPID program improvement was the result of an informal, ongoing process of feedback and correction. As a result, in general these RAPID programs were built and honed in real time, and the iterative process of assessment and correction served as the primary mechanism of program development. In other words, as explained by an interviewee: “we really learned by doing it.” (Innov KI01).

This process of iterative program development provided a channel for staff and providers to advocate for changes needed to create a better, more efficient and more sustainable program—such as adding linkage staff once data-on-demand for RAPID services became clear to leadership in the FQHC site, or expanding physical space to accommodate an increased volume of RAPID patients at the Testing Site.

“We’re building out staffing to be able to see more folks as they drop in—so having more nurse practitioners. [Also] we're expanding to the third floor to use one of the counseling rooms. I think we've already started using one of the counseling rooms up here for an additional like blood draw station. We're renovating our clinic to add another exam room on the second floor. So, there are like staffing and space needs that are being addressed to ideally build out that capacity.” (Test KI11)

This process also provided a framework in which those performing RAPID services could identify and address ways to improve the patient experience as observed from their close patient contact—i.e., creating access to rideshare transportation services to ensure patients had a reliable and safe transportation option to attend HIV-related appointments.

“We have UberHealth, which has been very helpful. Like we got one guy who we brought him up from San Jose. We brought him all the way up here. … He must’ve been acutely infected, so that means like they left here. Like in three days they get a positive viral load, and I’m like, he has no ride up here. Like what do you do? And so it’s a barrier, so then you solve it.” (Test KI04)

- (6) Patient-centered approach.

Implementation of RAPID programming in each site was a patient-centered endeavor. Not only was the well-being of the patient the primary motivation for implementing RAPID, but the content of every encounter was shaped around meeting the unique needs of a patient. For example, while providers laid out the rationale for starting ART, ultimately the decision to initiate treatment rested with the patient, as an interviewee from the Innovator Site explained:

“I think with managing any kind of chronic illness, you can be nervous for your patient. You're just hopeful that they're able to follow directions or keep track of their meds or keep coming to their scheduled appointments. So, for me, yes, I definitely had reservations, and you still move forward and try and support people the best you can. Because if they say they want to start meds, it would feel so inappropriate to deny them medication… I think that it's important to just respect what a patient wants after they're given non-biased, nonjudgmental information and support. They get to decide the next step. And then you help create a care plan that makes it as easy as possible.” (Innov KI05)

Even provider buy-in was patient-centered; some providers who initially harbored doubts about the same-day treatment model were convinced of its value once they saw the positive effect RAPID ART had on patients.

“Are we pressuring patients to do this? How is it really going to help them? I think fairly quickly we kind of got that from the patients…they really saw that as an opportunity to like take control over this virus that they thought was like out of control in their body. So, it was actually, actually pleasantly surprising. It was really a nice surprise because I was kind of worried.” (Innov KI03)

Common among the sites was the patient-centered goal of providing the patient with an interdisciplinary experience, whether or not RAPID services were executed by an interdisciplinary team.

“One of the very special things about this clinic but definitely the … RAPID team is how interdisciplinary it is and how none of it would work if anybody was missing. Like, that there needs to be a nurse, and there needs to be social workers, and there needs to be eligibility, and there needs to be providers, and, like, it's such a beautiful example of the power of interdisciplinary work.” (Innov KI04)

“We practice as a group here. That means if someone tests positive, uh, it has to be done by an NP. . . And the - the clinic pretty much steps up and kind of creates that space for that NP to spend the amount of time that they need with that person.” (Test KI04)

Participants from the Innovator Site talked about the power of metaphoric handholding and creating a “red carpet” experience for a patient. RAPID team members at all sites strategically guided patients through each aspect of the visit, including minimizing unescorted time and providing warm hand-offs between providers. When done well, warm hand-offs served to exemplify respect and trust between team members. Being around a dynamic of friendliness and comradery among RAPID team members can help a patient to feel more comfortable with the various clinic staff to whom they were being handed off.

“They get that it needs to be a red-carpet event, so the RAPID patient will meet every single member of the team. Like the provider, the social worker, the nurse. Even if they're not necessarily working with that person that day. It's just like, "Hey! I am the RAPID nurse," or, "I am the RAPID social worker and I am - we're not going to meet today, but we can meet next week, if you want." We're part of the same team, here to support you. We're happy that you're here. It's just reinforcing that they have a lot of people behind them. … That they do a really good job.” (Innov KI09)

At the Innovator Site, they aspired to meet with the patient to execute at least a portion of RAPID activities as a team. At the Testing Site, ideally just a nurse practitioner and health navigator would interact with the patient. Restricting the number of people involved in the RAPID ART visit minimized the number of times the patient would have to disclose their new HIV status. Other related activities, including medication procurement, benefits eligibility assessment, and lab processing were executed by staff behind the scenes. At the FQHC the RAPID process and order of operations was flexible and depended on what would make for the most smooth and efficient process for the patient:

“So, instead of somebody finding out their diagnosis and me meeting with them right away, they'll meet with a provider first, and then they'll meet with like a [partner] service person, and then they'll meet with me. So, it's just the structure is a little bit different depending on like which clinic the person is diagnosed at.” (FQHC KI03)

When possible, the RAPID protocol at the FQHC was adjusted to meet patient needs and per the feedback of staff who were on the ground observing where, when, and how clinic operations warranted adjustment. A provider explained a problem related to the undesirable situation of leaving a patient sitting alone while the linkage coordinator traveled from a different clinic to reach the patient:

“I don't have capacity to sit in the room for 30 minutes while we wait for someone to come. Our nursing team doesn't have that capacity. So a lot of times for a substantial amount of that period, the patient is sitting alone in an office exam room, living with that diagnosis. And I always thought that that was not ideal. A lot can happen in your head in 30 minutes in a room alone.” (FQHC KI05)

As a result of this type of observation, the FQHC shifted the order of who would meet with the patient to fill in the linkage specialist’s travel time and eventually added an in-house linkage person at this clinic.

- (7) Strong communication methods and culture.

RAPID required the coordination of numerous clinic staff to engage in multiple separate tasks simultaneously and quickly. While communication of some type is necessary to operate any program, effective communication methods reduced implementation challenges. For example, updating and disseminating explicit policies and protocols ensured that everyone involved in the RAPID process understood their roles. The extent to which a RAPID visit could be tailored to a patient’s specific needs was shaped in large part by the quality and efficiency of the communication between team members and across service areas.

Each site formalized RAPID communications differently. The Innovator Site and the FQHC employed a single point of contact for new RAPID referrals (one used a pager system, the other used a RAPID phone staffed by linkage coordinators). The Testing Site operated within a compact space and when a client was diagnosed with HIV, the test counselor would alert the lead clinician to initiate the RAPID process. Given the small size of the facility and the close proximity of providers and staff to one another, close collaboration and open communication among providers and staff were hallmarks of the Testing Site clinic culture.

Key informants also provided examples of communication breakdown, further highlighting, by contrast, the importance of this element. For example, early episodes of fractured communication across service areas disrupted implementation roll-out:

“[Some of the] team actually wasn't even aware that we were trying to launch [RAPID], because they weren't really present [on-site]. They were primarily at the [other] site. And I think there was just bad communication happening at that time.” (Test KI11)

At the FQHC, one informant described how the persistent ineffective communication of protocol changes in one area not only troubled RAPID operation, but presented a real danger to the sustainability of the program through damage to staff acceptability:

“They change things, and then they really don't give us directions on the next steps. They changed [to a new pharmacy software] and then didn't really train us on how to [use it]. … It's little things like that that kind of happen frequently, with other examples. So, let's see. They changed ADAP on us. They just tell us things at the very, very, very last minute, and like the transparency is just like – by the time it gets to us, like we're already with a patient. And then it's just like, okay. And then you just have to figure it out as you go…” (FQHC KI01)

This informant went on to attribute staff turnover to these “little things.” Thus, determining effective communication strategies or course correcting when it was clear that communication was ineffective was critical for successful RAPID implementation.

### Challenges to implementation with implications for sustainability

Notable in these interviews was the duality of successful RAPID implementation along with stories of addressing challenges. While some informants insisted that RAPID was “easy,” they also told stories of having to deal with transitional tension.

“For me as a clinician, [RAPID ART] was very easy, and it was a wonderful thing that we were able to offer it. So, for me, it was easy, logical, organic.” (Test KI05)

“I think as time goes on, it's easier to manage and kind of predict, but in the beginning, that was a pretty tough transition.” (FQHC KI03)

Tensions described by interviewees related to the transitions associated with RAPID program implementation, provider strain and burnout, the intensity of increased patient volume, and the stress of modifying RAPID processes to address changing needs over time. We offer these findings as a set of common challenges that may be more easily managed if they are expected.

#### Transitional tension

Even for staff who were eager to be part of providing RAPID services, the transition to new processes—including changes to schedules and workflow, and training and cross-training in new skills and duties—was a challenge. Training in the organization’s RAPID protocol, medication access, and related benefits required time and energy, and was typically added on top of every-day duties. Cross-training, where staff who already possessed required competencies trained other team members, was an effective strategy to distribute RAPID duties more evenly across team members, but the process of cross-training could add strain to the individual responsible for conducting said training as well. For example, a PrEP navigator working at the Testing Site leveraged their expertise in medication access and eligibility assessment to occasionally assist RAPID clients. That PrEP navigator trained other staff in these skills which temporarily strained the PrEP navigator who was also juggling a full load of PrEP navigation duties.

“And then while [the cross-training] process is happening, managing the folks who are holding the work at that same time. You know, like we're still having to do benefits, we're still having to do, um, medication acquisition while the other team is being trained.” (Test KI11)

In some cases, transitional challenges were experienced as pain points that would eventually resolve (i.e., staffing or space reconfigurations), though not in all cases. Some informants reported instances of transition-related turnover, where occasionally a staff member would not be a good fit for the new competencies required by RAPID processes, or a change to staffing schedules was not acceptable to a person in an impacted role and consequently, as highlighted earlier, they sought work outside of the organization.

“Assuming they're interested [in cross-training], um, then it becomes, are they going to be able to do the work? [There are] different learning curves that come with [new skills]. So, are people going to be able to do the work that we're kind of tasking them with…?” (Test KI11)

The acute transition stage of implementation/iteration was typically a tense period, though multiple participants reported that the difficulty was temporary and improved over time. Thus, interpersonal friction and individual frustration can be anticipated, are transitional, and are not necessarily a sign that a RAPID program is not feasible or sustainable at a particular site.

“Right now it’s painful with all these transition periods, as we build out staff and training the staff. But the idea is that we’ll be able to see folks without kind of derailing the clinic flow as much as it currently is and whenever certain situations pop up. So, that definitely seems like it’s painful for now, but we’re able to get through it.” (Test KI11)

#### Burnout/provider strain

Data from interviews showed high levels of buy-in for RAPID and that the implementation process was sustainable up to a point; sustainability became problematic when staff and provider strain threatened to lead to burnout. Staff who did not feel sufficiently supported or were already overtaxed prior to RAPID implementation were at particular risk of burnout. One prescribing provider who took a lot of pride in their work and their connection with their clients talked about both appreciating the emotional authenticity and engagement of the RAPID interaction, but also wished that there was an opportunity, “even 15 min” to decompress (Test KI02), maybe take a walk around the block, before returning to seeing other patients. Another interviewee explained the strain of balancing the duties of engaging in a RAPID encounter against the work of caring for their existing client caseload.

“This is just the same-day start in general. Like when they implement these programs, they should talk to the people who are going to be doing it for more input, because these people are getting burnt out really quickly. … they're just burnt out, like tired. And then not only are they doing same-day start, but they have a full caseload of 40 individuals that are newly positive that they have to retain and engage in care. So, it's not just a same-day start, and then that's it. Your job is not done. You have to maintain contact with these people, schedule their monthly or bimonthly medical appointments, meet them at their appointment, try to get them to come to support groups, be their sometimes sole supporter, make sure they have their medication, check in on them, see how they're doing, make sure they're taking their medication. If they're not taking their medication, see what the problem is. Like it's way more.” (FQHC KI01)

#### Volume

The number of RAPID visits a site executed over a period of time, or RAPID volume, was reported as a high-impact factor in program sustainability. A primary constraint that clinics came up against was that of capacity: “how many [RAPID encounters] can you do?” (Test KI04) Even the most seamless RAPID program may have limits; a program designed to absorb the disruption of a RAPID visit that sees one RAPID case a month is a fundamentally different program than one that sees one a week, or one a day.

“Originally I thought we were going to be doing 12 a year. …. So, that was my initial proposal for it. And then when all these people came in, I think we did like 65 last year. I’m like, that’s more than 12.” (Test KI04)

“During the next year, 2017–2018, it became increasingly less manageable. The numbers were growing, our staff was not.” (Test KI03)

Both adopter sites struggled with an unanticipated increase in volume as their RAPID programs took root. The Testing Site began receiving increased referrals as other agencies learned of their success with RAPID, while program success led to overall growth at the FQHC, outpacing provider capacity:

“[The RAPID program we implemented] was just meant to be for our folks. And then what was happening is people were testing positive at other locations, and that location couldn’t take care of them, so they were funneling them to us. You know, so we kind of became this referral center.” (Test KI04)

“One of the difficult things we're now facing is that [our site] and same-day start is growing so rapidly that like just follow-up appointments are really difficult. So, in the beginning, you were supposed to have a follow-up appointment one to two weeks out from diagnosis. But now, it's like a month to a month and a half just because all of the providers are so booked, and a lot of them don't even have open panels [and] scheduling that initial follow-up is so difficult. And then if somebody misses that follow-up appointment, we're not seeing them for like three months after their initial diagnosis just because scheduling is so hard.” (FQHC KI03)

#### Needs may change over time

Related to the need for adaptive capacity, the needs of a RAPID program evolved over time. Sites ended up needing more staff, needing to change clinic flow, and needing more physical space, for example. Additionally, future ways of immediately accessing ART medications were raised as another area likely to change. One site recognized as their program progressed that the mental health needs that arose for some of their patients as part of the diagnosis and disclosure experience would be best served by in-house mental health services. Another site recognized that RAPID as a program could better serve patients if some key staff worked four 10-h shifts in a week rather than five 8-h shifts. Some changes arose from iterative quality improvement activities as clinics sought to optimize services and patient and provider experiences, while, in the future, some changing needs may be driven by conditions such as funding fluctuations or COVID-19 restrictions.

## Discussion

RAPID implementation relies less on the topical application of new elements onto an existing clinic structure than it does on the coordination of a clinic’s existing human resources and the cultivation of systems and processes that support unpredictable needs and patient-centered care. Practical application of the seven essential elements described above means: identifying where these elements currently exist within a practice; leveraging them when possible; strengthening them when necessary or developing them if they do not yet exist; and looking to these elements when challenges arise for potential solutions.

Reports of success and ease with the provision of RAPID services were attributed, either directly or indirectly, to strong communication, high adaptive capacity, and a commitment to patient-centered care, while many of the challenges relayed by informants reflected situations in which flexibility had reached a limit or communication had broken down. Our finding that these elements are the essential building blocks of a successful RAPID program should not be interpreted to mean that these elements were wholly present or executed perfectly at each site. Indeed, identifying instances where a weak or lacking component posed a threat to a program was as informative as identifying the inverse.

It is important to note that the operational structure of RAPID programs is shaped by the clinical setting in which they function, and therefore the essential elements identified herein will not apply equally to all programs. Some settings have onsite primary care, which reduces or may eliminate the role of linkage, compared to settings that have to link patients to external primary HIV care. Some settings receive newly diagnosed patients as referrals from outside their system while others do not—a factor that can affect the volume of RAPID patients. Evidence suggests that, in the United States, settings with existing HIV testing programs or strong referral partnerships between testing and clinic entities may be in a position to implement a RAPID program without adding significant resources [11, 17]. In locations with weak HIV testing and ART delivery infrastructure and limited human resources, implementation of RAPID programming may be less feasible.

The goal of this analysis was to identify essential elements of RAPID programs—components that would ideally be present for successful implementation of RAPID. These elements are similar to, but distinct from, facilitators and best practices. The ‘implementation champion’ was reported on as an essential element here, however moving forward it may prove to be a nice, but not necessary element. The literature suggest and our data support the importance of a champion in facilitating the successful implementation of a novel intervention; this may be especially important during the innovation and early adoption phases of diffusion of an innovation. Once an innovation becomes more mainstream, the role of the champion becomes less critical. Future RAPID implementers will need to determine whether their setting warrants identification or cultivation of a RAPID champion. Relatedly, champions often serve to build buy-in for an innovation. While we briefly described provider buy-in as a key component of the comfort and competence to prescribe RAPID ART, we take up this concept more fully in a forthcoming publication. We recognize that while identifying providers with knowledge of HIV treatment was not an issue at any of the sites participating in this study, given the national shortage of HIV providers, it could be a problem in other clinic settings. [30, 31].

This study has limits. The sites selected for analysis are highly skilled in providing culturally competent care to people living with HIV. Our findings may be less transferable outside of those contexts. Our sample from the FQHC site was smaller relative to the other sites, and notably more potential participants declined to participate in that site than the others. This could have been because providers at this site were less experienced with RAPID services and may have felt they would not be able to meaningfully contribute to the study. Providers at this site may have been too busy to prioritize participation in a research study. Finally, the geographic difference and weaker ties between the research team and the prospective participants in the FQHC site may have been a further deterrent to participation.

Future research should provide insights into how the new paradigm of RAPID ART becomes normalized in a clinical setting. Such research should also be improved upon with the addition of a more granular, ground-level exploration of how individual providers approach the RAPID interaction with patients.

## Conclusion

Based on extensive interviews with RAPID staff, we provide evidence that the RAPID model can be applied to a diverse range of clinical contexts with a higher degree of provider/staff acceptability than was previously known, and a synthesis of the essential elements necessary to do so. These essential elements are not inclusive of all facilitators identified in this study; rather they are the essential elements common across sites, without which the programs may not have been able to operate. These essential elements may be easily achieved in some clinical settings—such as those supported by the Ryan White HIV/AIDS Program—and difficult to achieve in other settings without, for example, a willing prescriber or a reliable payer source to cover the costs of medication, labs, and clinic visits. We are optimistic about future development of RAPID programming throughout the U.S. and hope future implementers of RAPID will take inspiration from the examples of the three successful programs outlined here to create a program consisting of some or all of these essential elements, and work towards developing the ‘ingredients’ that may not yet exist in their environments.

## Supplementary Information

**Additional file 1.** Interview Guide.

**Additional file 2.** CFIR Map.

## Data Availability

The dataset used and/or analyzed during the current study are available from the corresponding author on reasonable request.

## Abbreviations

ADAP: AIDS drug assistance program
APN: Advanced practice nurse
ART: Antiretroviral therapy
CFIR: Consolidated Framework for Implementation Research
DO: Doctor of osteopathic medicine
FQHC: Federally qualified health center
HE: Health educator
LTC: Linkage to care coordinator
MD: Medical doctor
MSW: Master of social work
NP: Nurse practitioner
PA: Physician’s assistant
RN: Registered nurse
