# Supplementary material for: Essential elements of and challenges to rapid ART implementation: a qualitative study of three programs in the United States
Source: BMC Infect Dis. 2022 Mar 31;22:316. doi: 10.1186/s12879-022-07297-3 (PMC8968260; doi:10.1186/s12879-022-07297-3)
Supplement: Supplementary file 1 — Additional file 1. Interview Guide. [file 12879_2022_7297_MOESM1_ESM.docx]

**Opening statements**

As we told you when we first spoke, we are doing a study about provider perspectives on the RAPID linkage and ART initiation model. Your participation is very important because we want to learn about your interactions with the clients who have gone through – or not gone through – RAPID linkage and ART initiation. We are also interested in hearing about the strengths of the initiative and what, if anything, could be improved. We expect that this interview will last about 30-60 minutes.

Everything that you tell me will be confidential, except for things that are reportable by California state law, such as the intent to harm yourself or others, child abuse, and elder abuse. I may make some notes as we talk in order for me to remember questions that I want to ask you later. Also, I will record the interview so that we can analyze what was said later. If you wish to not answer a question at any time, please say so and we will move on. If you wish to end the interview for any reason, please let me know and we will end immediately. This won’t affect your receiving the monetary incentive that we offered. If you need to attend to urgent work issues or need to take a break for a few minutes, please let me know and we can take a short break.

# Consent Procedures and Confidentiality

Here is a consent form about the study. Would you like me to read it to you or would you like to read it yourself? *(Give participant the consent form and a copy of the bill of rights. Go through each section to make sure individual understands the form. Identify the P.I. and the P.I.’s contact information.)* I am happy to answer any questions about this consent form or about the study. If you agree to participate, we will obtain signed written consent from you.

The audio-recordings and transcripts of your interview will not have your name or any information that can identify you. If you mention other people during the interview, we can delete or change their names in the transcript of our conversation.

Before we start the interview, I would like to ask you to fill out a brief questionnaire with some basic information about yourself. This information will not have your name on it either.

**–** *BEGIN AUDIO-RECORDING*

| **Questions** | **CFIR query domains / constructs** |
| --- | --- |
| **Introduction** |  |
| *Most of our questions will be open-ended and are designed to solicit your thoughts/experiences so please do not hesitate to share whatever you believe might be related to any of the topics.*  *I would like to ask you a few questions to help me understand your role and how you are involved in the RAPID Program at your site.* |  |
| 1. How long have you been practicing HIV medicine? |  |
| 1. How long have you been working/practicing in this clinic? |  |
|  |  |
| 1. What is your role in RAPID?    1. How long have you been in this role?    2. Has your role changed at all over time? | Background information |
| 1. Were you involved in the initial implementation of the RAPID initiative? | **Intervention Characteristics**  Intervention Sources |
| **Implementation Experiences** |  |
| *I would like to hear more about how the process of implementing RAPID linkage and same-day ART initiation, and how you and others viewed the idea before it was implemented.* |  |
| 1. What, if any, other initiatives have [site] undertaken that have helped to pave the way for RAPID? 2. Tell me about the process of implementing the RAPID approach.    1. On a scale of 0-10, with 0 being very easy and 10 being nearly impossible, how difficult was it to implement RAPID?    2. What were the barriers?       1. Constraints? I.e., financial support, staff time and expertise and/or competing priorities?    3. What were the facilitators?    4. What role has organizational culture played in implementing RAPID? | **Inner Setting**  Readiness  **Intervention Characteristics** Evidence Strength/Quality  **Implementation Process** |
| 1. Please describe any changes in clinic flow and policy that had to be made for RAPID. 2. What kind of support have you received from your leadership? | **Inner Setting**  Leadership Engagement |
|  |  |
| **Attitudes Towards RAPID/Operational Experience** |  |
| 1. What do you think about the RAPID approach? |  |
| 1. How do you think the RAPID program meets the needs of clients? 2. How well do you think the RAPID program meets the treatment goals you and your clients/clients have discussed? | **Individual Characteristics** Knowledge & Beliefs |
| 1. What do you think the degree of acceptance of the RAPID approach is by the people involved in it?    1. Why is that?    2. What do you think the degree of acceptance of the RAPID approach is by the people not directly involved in it?    3. What would you say is the degree of acceptance from leadership? | **Inner Setting**  Implementation Climate; Compatibility |
| 1. What specialties and external parties are involved with RAPID?    1. How do they complement each other?    2. Are there any redundancies?    3. Is any specialty or ancillary service missing? | **Outer Setting**  Cosmopolitanism;  External Policies |
| 1. Do you feel that there is flexibility in the RAPID approach? | **Intervention Characteristics** Adaptability |
| 1. Are there aspects of service delivery that require tailoring? How do you accomplish that? | **Inner Setting**  Available Resources |
|  |  |
|  |  |
| **Testing and Referral** |  |
| 1. Let’s shift gears a little bit. Tell me about how you discuss HIV test results with a client. What are some common questions clients have? How do you address these questions? | **Outer Setting**  Patient Needs & Resources |
| 1. How are clients referred to RAPID? What makes a clinic site a good partner with the RAPID program? | **Inner Setting**  Networks & Communication; Compatibility |
| 1. If the RAPID model were expanded to other testing sites, what are the things that would make referrals to the RAPID program work well? |  |
| 1. Tell me about how you start the conversation with a RAPID client when you first meet them. If we were in the room with you, what would it look like? What would you say? | **Implementation Process** |
|  |  |
| **Antiretroviral Therapy** |  |
| 1. A main focus of RAPID is getting newly diagnosed individuals on ART as quickly as possible. Can you walk me through how you discuss ART with a RAPID client? What do you say? | **Implementation Process** |
| 1. What are the essential points that you feel need to be covered? 2. Do you talk about the biological processes of HIV? |  |
|  |  |
| 1. How do you assess client readiness to start ART? How do you weigh client preferences? | **Implementation Process** |
| - 1. How do you handle clients’ emotions? Do you ever feel clients are not ready? Tell me what that means to you.   2. *If participant can’t think of anyone who hasn’t been ready, or reports not needing to assess readiness*: is there someone you would be concerned about? | **Outer Setting**  Client Needs & Resources |
| 1. Do you ever refer RAPID clients to other resources for HIV and ART education? What kind of resources? | **Outer Setting**  Client Needs & Resources; Cosmopolitanism |
| 1. If a RAPID client agrees to start ART, how do you begin treatment? Walk me through what happens. | **Implementation Process** |
| - 1. What are the barriers to starting? Facilitators? |  |
| Tell me about some of the RAPID clients you have had who have declined immediate ART start. What was going on for them? Did they start eventually? Why or why not? | **Outer Setting**  Client Needs & Resources |
| Can you tell me about your understanding of why they declined? And if they started eventually? |  |
| 1. What kind of adherence support is available to RAPID clients? | **Inner Setting**  Available Resources |
| 1. **IF APPLICABLE**: I understand that in this program, clients are offered treatment here at [site] and then are typically linked to a new care site. How is that process for you? What is it like to start a client down the path and then hand-off? |  |
| - 1. What kind of concerns do you have when you link a client to a care site?   2. What type of communication happens once a client is linked? |  |
| **Client Experience** |  |
| 1. Walk me through a recent experience working with a RAPID client. How typical was this case? |  |
| 1. Tell me about the most challenging situation you’ve encountered with a client. | **Implementation Process** |
| 1. Tell me about a client you consider to be a success story. 2. In what ways are clients who have gone through RAPID different from clients who have not?   *The following are de-prioritized questions due to time limitations and potential for redundancy. Ask only if these issues did not come up organically in prior responses.* | **Intervention Characteristics**  Relative Advantage |
| 1. How do you talk about disclosing to partners? Family? Others? |  |
| How often do you address the topic of disclosure to partners/friends? Can you describe that to me? |  |
| 1. How does HIV stigma impact your first encounter with a RAPID client? How do you talk about it? 2. What other challenges do clients face that haven’t already been mentioned? Are there other kinds of supports you think RAPID clients could benefit from? What things are particularly destabilizing? Are there “red flags” that indicated a client might not do well in the RAPID model? |  |
| **Level of Endorsement and Recommendations** |  |
|  |  |
| 1. On a scale of 0-10, how successful do you think the RAPID Program is at your site? Why?   Note: We are interested in the “perception” of success; the interviewee can define success in any way   1. In what ways could RAPID be improved or augmented?   OR: What are the gaps between the way the program operates now and how it might operate more optimally in the future? | **Implementation Process**  Executing  **Implementation Process**  Reflecting and Evaluating |
| 1. Do you have any concerns moving forward? |  |
| 1. Would you recommend that other sites implement RAPID? What specific suggestions do you have for sites that are seeking to implement RAPID? |  |
|  |  |
| 1. Is there anything else you would like to talk about that we haven’t already discussed? Do you have any questions for me? |  |
| **Thank you for participating.**  **–** *ADMINISTER DEMOGRAPHIC QUESTIONNAIRE*  Thank interviewee for his/her/their time and for participating in the study. Give participant the monetary incentive. |  |
