# Supplementary material for: Essential elements of and challenges to rapid ART implementation: a qualitative study of three programs in the United States
Source: BMC Infect Dis. 2022 Mar 31;22:316. doi: 10.1186/s12879-022-07297-3 (PMC8968260; doi:10.1186/s12879-022-07297-3)
Supplement: Supplementary file 2 — Additional file 2. CFIR Map. [file 12879_2022_7297_MOESM2_ESM.docx]

| **CFIR domain/construct** | **Construct description** | **Interview query** | **Finding** |
| --- | --- | --- | --- |
| **Intervention Characteristics** | | | |
| Intervention Sources | Perception of key stakeholders about whether the intervention is externally or internally developed. | Q. 4 |  |
| Relative Advantage | Stakeholders’ perception of the advantage of implementing the intervention versus an alternative solution. | Q. 25-26 |  |
| Evidence Strength / Quality | Stakeholders’ perceptions of the quality and validity of evidence supporting the belief that the intervention will have desired outcomes. | Q. 6 | Finding 1: Presence of an implementation champion |
| **Outer setting** | | | |
| External policies and incentives | A broad construct that includes external strategies to spread interventions, including policy and regulations (governmental or other central entity), external mandates, recommendations and guidelines, pay-for-performance, collaboratives, and public or benchmark reporting. | Q.11 | Finding 3: Expedited access to ART medications |
| Cosmopolitanism | The degree to which an organization is networked with other external organizations | Q. 11, 18 | Finding 3: Expedited access to ART medications |
| Patient needs and resources | The extent to which patient needs, as well as barriers and facilitators to meet those needs, are accurately known and prioritized by the organization. | Q. 9, 13, 17, 18, 20 | Finding 5: RAPID team member flexibility and organizations’ adaptive capacity  Finding 6: Patient-centered approach |
| **Inner setting** | |  |  |
| Structural Characteristics | The social architecture, age, maturity, and size of an organization. |  | Finding 5: RAPID team member flexibility and organizations’ adaptive capacity |
| Networks and communication | The nature and quality of webs of social networks and the nature and quality of formal and informal communications within an organization. | Q. 14 | Finding 7: Strong communication methods and culture |
| Compatibility | The degree of tangible fit between meaning and values attached to the intervention by involved individuals, how those align with individuals’ own norms, values, and perceived risks and needs, and how the intervention fits with existing workflows and systems. | Q. 10, 14 | Finding 6: Patient-centered approach |
| Culture | Norms, values, and basic assumptions of a given organization. | Q. 6(d) | Finding 5: RAPID team member flexibility and organizations’ adaptive capacity  Finding 6: Patient-centered approach  Finding 7: Strong communication methods and culture |
| Implementation climate | The absorptive capacity for change, shared receptivity of involved individuals to an intervention, and the extent to which use of that intervention will be rewarded, supported, and expected within their organization. | Q. 10 | Finding 5: RAPID team member flexibility and organizations’ adaptive capacity  Finding 6: Patient-centered approach |
| Access to knowledge and information | Ease of access to digestible information and knowledge about the intervention and how to incorporate it into work tasks. |  | Finding 5: RAPID team member flexibility and organizations’ adaptive capacity |
| Available resources | The level of resources dedicated for implementation and on-going operations, including money, training, education, physical space, and time. | Q. 12, 21 | Finding 4: Expertise in benefits, linkage, and care navigation |
| Goals and feedback | The degree to which goals are clearly communicated, acted upon, and fed back to staff, and alignment of that feedback with goals. |  | Finding 7: Strong communication methods and culture |
| **Characteristics of individuals** | |  |  |
| Knowledge and beliefs | Individuals’ attitudes toward and value placed on the intervention as well as familiarity with facts, truths, and principles related to the intervention. | Q. 9 | Finding 2: Comfort and competence prescribing RAPID ART |
| Self-efficacy | Individual belief in their own capabilities to execute courses of action to achieve implementation goals |  | Finding 2: Comfort and competence prescribing RAPID ART  Finding 4: Expertise in benefits, linkage, and care navigation |
| **Implementation process** | |  |  |
| Reflecting and evaluating | Quantitative and qualitative feedback about the progress and quality of implementation accompanied with regular personal and team debriefing about progress and experience. | Q. 32-34 | Finding 7: Strong communication methods and culture |
| Executing | Carrying out or accomplishing the implementation according to plan. | Q. 31 |  |
| Champions | Individuals who dedicate themselves to supporting, marketing, and ‘driving through’ an implementation; overcoming indifference or resistance that the intervention may provoke in an organization. |  | Finding 1: Presence of an implementation champion |
